# Research Progress of Fibroblast Growth Factor 21 in Fibrotic Diseases

**DOI:** 10.1155/2022/5042762

**Published:** 2022-05-29

**Authors:** Min-Qi Jia, Cha-Xiang Guan, Jia-Hao Tao, Yong Zhou

**Affiliations:** Department of Physiology, School of Basic Medical Science, Central South University, Changsha, Hunan 410078, China

## Abstract

Fibrosis is a common pathological outcome of chronic injuries, characterized by excessive deposition of extracellular matrix components in organs, as seen in most chronic inflammatory diseases. At present, there is an increasing tendency of the morbidity and mortality of diseases caused by fibrosis, but the treatment measures for fibrosis are still limited. Fibroblast growth factor 21 (FGF21) belongs to the FGF19 subfamily, which also has the name endocrine FGFs because of their endocrine manner. In recent years, it has been found that plasma FGF21 level is significantly correlated with fibrosis progression. Furthermore, there is evidence that FGF21 has a pronounced antifibrotic effect in a variety of fibrotic diseases. This review summarizes the biological effects of FGF21 and discusses what is currently known about this factor and fibrosis disease, highlighting emerging insights that warrant further research.

## 1. Introduction

Fibrosis denotes a disease of abnormal repair and excessive scarring, which is characterized by excessive accumulation of extracellular matrix (ECM). It can lead to tissue and organ structure remodeling [1]. Due to its high morbidity and lacking effective treatment, fibrosis disease contributes to a large amount of death [2–5]. Since its broad impact and serious consequences, fibrosis has become one of the main global healthcare issues [6]. Still, therapeutic strategies targeting the pathogenesis of fibrosis remain scarce.

Fibroblast growth factor 21 (FGF21) is a member of the FGF19 subfamily. FGF21 has multiple biological effects, for instance, anti-inflammation [7], antiapoptosis [8], antifibrosis [9], prevention of oxidative stress [10], and prevention of vascular calcification [11]. Thus, FGF21 is a vital endogenous protective factor in the body. Among existing studies, the best-known function of FGF21 is a promising pleiotropic regulator in glucose and lipid metabolism [10]. An increasing number of biological functions of FGF21 have been revealed in other studies and reviews minutely. In recent years, the role of FGF21 in fibrosis has aroused researchers' concern. Fresh evidence supported that FGF21 weakens pulmonary fibrosis by alleviating oxidative stress *in vivo* and *in vitro* [10]. FGF21 ameliorates pancreatic fibrogenesis by regulating the polarization of macrophages [12]. It also protects the heart from angiotensin II- (Ang II-) induced cardiac hypertrophy, remodeling, and dysfunction *via* SIRT1 [13]. Targeting the FGF21/FGFR/*β*-Klotho pathway may halt or reverse hepatic fat infiltration, inflammation, and fibrosis [14]. Studies investigating FGF21 in the context of fibrosis have virtually exploded in the last decade. This review will focus on discussing published studies on the progression of FGF21 and fibrotic diseases.

## 2. Overview of Fibrosis Diseases

It is generally believed that the cause of fibrosis is chronic and sustained tissue damage. In different organs and tissues, various triggers can promote the progressive development of fibrotic diseases, such as genetic diseases, persistent infection, repeated exposure to toxins, irritants or fumes, chronic autoimmune inflammation, myocardial infarction, high serum cholesterol, obesity, poorly controlled diabetes, and high blood pressure [6, 15]. Fibrosis involves the occurrence and development of chronic inflammatory and autoimmune diseases. Besides, it affects tumor invasion and metastasis, chronic graft rejection, and the onset of many progressive myopathies [16]. If severely progressed in the end stage of the fibrotic process, the outcome is organ malfunction and ultimately leads to death [17]. Fibrosis can take place in almost every organ in the human body, as seen in liver cirrhosis, end stage of chronic kidney disease (CKD), idiopathic pulmonary fibrosis (IPF), diabetic cardiomyopathy, chronic pancreatitis, and some chronic autoimmune diseases [16, 18, 19]. Due to its high morbidity and lacking effective treatment, fibrosis disease contributes to a large amount of death [2–5]. Since its broad impact and serious consequences, fibrosis has become one of the leading global healthcare issues [6]. Despite a host of promising experimental data in the animal model, there are very few approved antifibrotic drugs that show significant effects on ameliorating or delaying the disease progression [20–22].

### 2.1. The Pathological Process of Fibrosis

**Figure 1:**
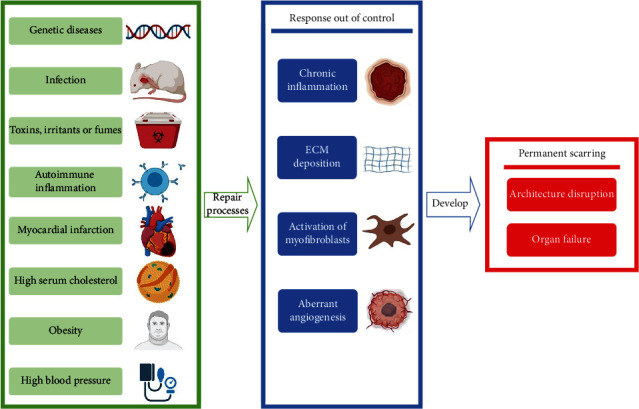
The pathological process of fibrosis. The pathological process of fibrosis can be divided into three stages. Chronic injury triggers tissue repair, which is later out of control, leading to an irreversible fibrosis reaction. This process ends up with permanent scarring and causes fatal consequences. Green box: etiological factors of fibrosis. Blue box: a common mechanism of the repair process. Red box: ultimate results of fibrosis.

The pathological process of fibrosis can be divided into the following stages (Figure 1). The initiation factor of fibrosis is persistent injury of tissue and organ [6, 23], such as chronic inflammation [24]. The body then activates a series of repair processes, including inflammation and the secretion of large amounts of ECM [25], to restore homeostasis rapidly. If the tissue injury is severe or the wound healing response is out of control, the tissue repair evolves into an irreversible fibrosis. Excessive accumulation of ECM components then occurs [26]. Ultimately, permanent scarring appears and finally develops into organ failure and eventually death [26, 27]. During fibrosis progression, inflammation and immune mediators such as cytokines, chemokines, and free radicals attempt to eliminate stimulating factors while activating quiescent fibroblasts into myofibroblasts to coordinate angiogenesis and the production of ECM components and promote tissue repair [28]. Whatever the trigger is, activation of myofibroblasts that produce ECM is central to all fibrotic diseases. Activated myofibroblasts are the core driver of fibrosis progression and ECM deposition [6].

### 2.2. Research Progress of Antifibrotic Mechanisms

**Figure 2:**
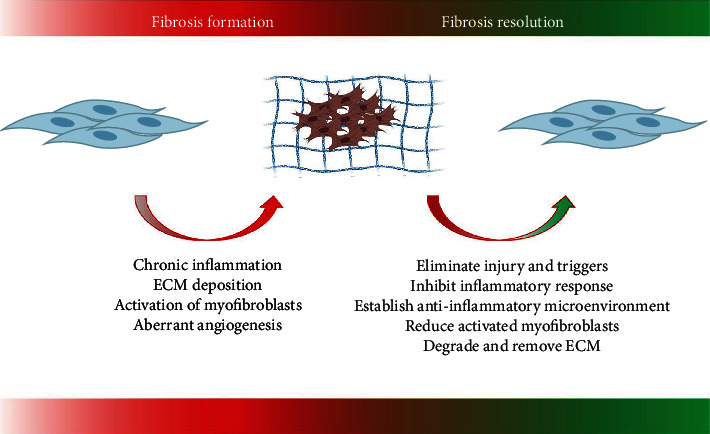
Antifibrotic strategies. Based on the mechanism of fibrosis formation, researchers have found several methods of promoting fibrosis resolution.

The most fatal fibrotic diseases are irreversible [2]. However, in some types of fibrotic disease, fibrosis is self-resolving and reversible. The balance between controlled fibrosis and the excessive scar is regulated by diverse approaches, molecules, and systems, mainly depending on the duration and repeatability of the injury. In the existing studies, the regression of fibrosis mostly has the following ways (Figure 2):

1. Withdrawal of the injurious stimulus and triggers: strong evidence from rodent models and human studies suggests that liver fibrosis is reversible when the factors that cause long-term and chronic damage to organ tissue are eliminated [29]
2. Inhibition of inflammatory response and anti-inflammatory microenvironment establishment [30]: phenotypic changes in immune cells involved in the inflammatory response affect fibrosis progression [29, 31, 32]. Inflammatory myeloid cells have a role in fibrosis [33–35]
3. Reduction of activated myofibroblasts: through apoptosis, dedifferentiation, transdifferentiation, etc., activated myofibroblasts revert to a static phenotype, thus preventing fibrosis progression [36–39]. Since activated myofibroblasts are the core driver of fibrosis progression and ECM deposition, a bulk of experiments have focused on the treatment of fibrotic diseases by regulating myofibroblast status in recent years [40–43]
4. ECM degradation: degradation and removal of ECM from tissues are crucial for the resolution of fibrosis [44]. Activation of matrix metalloproteinases (MMPs) and reduction of tissue inhibitors of MMPs (TIMP) resolve collagen and other ECM proteins [44]. Lysyl oxidase family members lysyl oxidase and lysyl oxidase-like proteins are extracellular copper-dependent enzymes that play a crucial role in ECM cross-linking, which are gradually recognized as a vital factor affecting the degradation of ECM [45, 46]

To sum up, currently, researchers have studied the progression and resolution of fibrosis from various perspectives. It is indispensable to understand the pathways governing fibrosis and identify therapeutic targets in these pathways for the development of new antifibrosis therapies for fibrotic diseases.

## 3. FGF21

**Figure 3:**
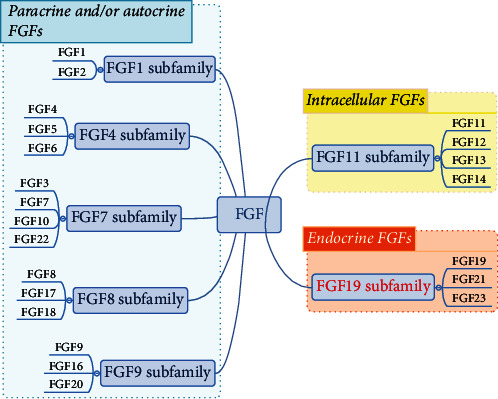
FGF family and endocrine FGFs. FGFs are grouped into seven subfamilies based on their differences in sequence homology and phylogeny. Blue-dashed box: paracrine and/or autocrine FGFs. Yellow-dashed box: intracellular FGFs. Red-dashed box: endocrine FGFs.

FGFs consist of a large family of proteins with significant effects on metabolism, organogenesis, embryonic development, and wound healing [47]. With an irreplaceable role in the metabolism of organs such as the liver, kidney, pancreas, brain, intestine, lung, and adipose tissue, FGFs have become one of the hotspots in the field of metabolism in recent years [48]. As shown in Figure 3, mammalian FGFs are divided into seven subfamilies, with a total of 22 species [49]: FGF1 and FGF2 belong to the FGF1 family; FGF3, FGF7, FGF10, and FGF22 constitute the FGF7 family; FGF4, FGF5, and FGF6 make up FGF4 family; FGF8, FGF17, and FGF18 are part of FGF8 family; FGF9, FGF16, and FGF20 are component of FGF9 family; FGF11 family consists of FGF11, FGF12, FGF13, and FGF14; FGF19 family comprises FGF19, FGF21, and FGF23 [50]. The classification standards are their differences in sequence homology and phylogeny. As their most common working pattern, FGFs are generally recognized as paracrine factors. In particular, some paracrine subfamilies are known for their functions in tissue patterning and organogenesis during embryogenesis: the first five subfamilies belong to this category [50]. The specific receptors of FGFs are coded by four genes (*FGFR1*, *FGFR2*, *FGFR3*, and *FGFR4*) and *FGFRL1* [48, 51]. Only through binding and activating specific high-affinity tyrosine kinase receptors can FGFs fully leverage the role of a pleiotropic regulator [52]. Due to its strong effect on regulating cellular lineage commitment, proliferation, differentiation, and apoptosis of various types of cells, growing researches suggest that the FGF/FGFR signaling plays a crucial role in masses of embryonic development processes and participates in the regulation of adult homeostasis [48, 53]. In contrast, the FGF19 subfamily recently shows its unique endocrine functional manner. As a foundation of an intact signal pathway, the presence of klotho protein in target tissues assists FGFs of the FGF19 subfamily to regulate bile acid, vitamin D, cholesterol, glucose, and phosphate homeostasis [50, 54]. FGF19- and FGF21-related pathways were activated under different physiological and pathological conditions [55].

The FGF19 subfamily includes growth factors that circulate through the blood, known as endocrine FGFs. By combining with FGFR, representatives of the FGF19 subclass exert their powerful effects [55]. FGF19, FGF21, and FGF23 show activity on FGFR1c, FGFR2c, FGFR3c, and FGFR4, while they cannot bind to FGFR1b, FGFR2b, and FGFR3b [55]. Compared with FGFs of other subfamilies, the FGF19 subfamily lacks a heparin-binding domain, which endows them a weak affinity for heparan sulfate [56]. With this feature, they can quickly spread from the site of secretion into the bloodstream and consequently work as hormones regulating distant target cells.

**Figure 4:**
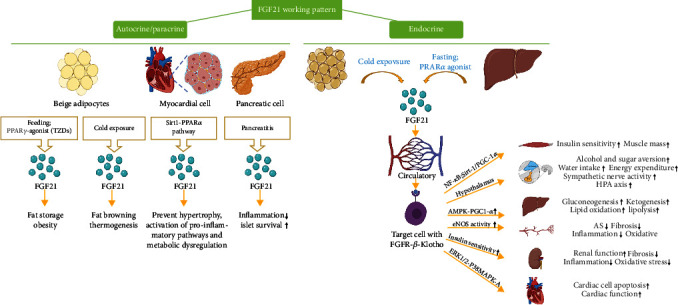
FGF21 working pattern. FGF21 is unique because of its possession of endocrine and autocrine/paracrine working manner simultaneously. Autocrine/paracrine manner plays an essential role in beige adipocytes, myocardial cells, and pancreas. They release FGF21 and have a biological effect on themselves to regulate metabolism and homeostasis. The liver and brown adipocytes are the primary producers of endocrine FGF21 in response to a broad spectrum of stress conditions. The circulating FGF21 finally reaches the target cells with FGFR-*β*-Klotho and exerts biological effects. FGF21 in blood circulation drives multiple signal axes in numerous tissues/organs, resulting in multifaceted beneficiary metabolic effects, including promoting (upward arrow) gluconeogenesis, ketogenesis, lipid oxidation, lipolysis, alcohol, sugar aversion, water intake, energy expenditure, sympathetic nerve activity, HPA axis, insulin sensitivity, muscle mass, cardiac cell apoptosis, cardiac function, and renal function and preventing (downward arrow) AS, fibrosis, inflammation, and oxidative stress.

FGF21, the factor we are interested in, is a member of the FGF19 subfamily. It was in mice that the *fgf21* gene first been discovered [55]. While searching for homologs, it was identified in the human genome [57]. Human FGF21 consists of 209 amino acids, the mouse protein of 210 amino acids with 75% sequence homology [58]. There are plenty of FGF21 synthesis sites: white adipose tissue, brown adipose tissue, pancreas, skeletal muscle, and cardiac endothelial cells, but the liver is the most vital [48, 59]. FGF21 diffuses into the blood from the secretory site and participates in the metabolic regulation of various tissues throughout the body [55] (Figure 4). Interestingly, FGF21 acts in an autocrine manner as well. FGF21 controls the appearance and activity of beige adipocytes through an autocrine manner [60]. One study proved that FGF21 produced by myocardial cells functions as an autocrine factor to attenuate metabolic disorder, prevent hypertrophy, and activate proinflammatory pathways in cardiac tissue [61]. In this study, the authors demonstrated that the SIRT1-PPAR*α* pathway upregulated *FGF21* gene expression [62]. In contrast to other fibroblast growth factors, the factors belonging to the FGF19 subfamily show a much lower affinity for FGFR, which decides that they require the presence of a coreceptor, Klotho proteins, to elicit activity on cells [63, 64]. Klotho proteins belong to a group of transmembrane proteins which consists of the following subfamilies: *α*-Klotho, *β*-Klotho, and *γ*-Klotho [55, 65]. *β*-Klotho mediates the biological effect of FGF21. Endocrine growth factors show a low affinity for the FGFR or Klotho protein itself but high for the FGFR-Klotho complexes. Since FGFR expression can be found in most tissues and cells, tissue-specific Klotho expression determines target tissues for the endocrine growth factors [66].

### 3.1. Physiology Functions of FGF21

In a healthy human body, FGF21 is highly expressed in the liver. It is released in response to high glucose, high free fatty acids, and low amino acid supply and regulates energy, glucose, and lipid homeostasis actions in the central nervous system (CNS) and the adipose tissue [67]. As a transcription factor, peroxisome proliferator-activated receptor *α* (PPAR*α*) controls FGF21 expression in the liver, which sequentially affects body lipid metabolism and energy homeostasis during starvation. Under fasting conditions, researchers notice a significant increase in FGF21 hepatic expression and FGF21 plasma concentration, which affects white adipose tissue and promotes adipose decomposition, oxidation of fatty acids in mitochondria, and browning of the white adipose tissue, as well as the appearance and activation of brown adipose tissue cells. Brown adipose tissue increases FGF21 synthesis and promotes energy metabolism in the body under cold conditions or the effect of norepinephrine [67–69]. Both of these belong to the endocrine modes of FGF21. Specifically, recent findings indicate that FGF21 also has an autocrine pattern in white adipose tissue, heart, and other tissues and organs [57].

FGF21 plays significant biological roles in the body. The most well-studied function of FGF21 is serving as a crucial multieffect regulator of glucose and lipid metabolism [57, 70, 71]. Kharitonenkov et al. have shown that FGF21 increases glucose uptake by mouse 3T3-L1 cells, which exhibit a phenotype similar to adipocytes under proper conditions [72]. In addition, induced by fasting/starvation, FGF21 increases glucose tolerance and tissue insulin sensitivity and lowers blood glucose levels [73]. Moreover, FGF21 promotes glucose [72], lipid uptake [74], and lipogenesis in adipose tissue [75], thereby preventing the accumulation of ectopic lipids in the liver and skeletal muscle. Another essential role that FGF21 plays is participating in the regulation of energy metabolism. In white adipose tissue, FGF21 stimulates glucose entry, regulates lipolysis, increases mitochondrial oxidative capacity, and enhances the effect of PPAR-*γ* through an AMP-activated protein kinase (AMPK)-sirtuin1-PGC-1*α*-dependent mechanism [76]. FGF21 increases glucose transport with glucose transporter-1 (Glut-1) instead of Glut-4 as insulin does [77]. Animal model studies indicate that *Fgf21* gene deletion (Fgf21-knockout, *Fgf21*-KO) [78] does not show PPAR*γ* effects, such as reduction of fat and lipemia, improvement of tissue insulin sensitivity, and increase of lipogenesis [55]. Apart from acting on the liver and adipose tissue, FGF21 is considered a key factor linking peripheral metabolic tissues and the brain to participate in the process of energy metabolism. Although FGF21 is not directly expressed in the CNS, it can diffuse across the blood-brain barrier in one direction and is present in the cerebrospinal fluid in humans [79]. Based on controlling of the neuropeptide corticotropin-releasing factor, FGF21 stimulates sympathetic nerve activity to brown fat tissue [80], thereby increasing energy consumption. Moreover, as FGF21 has been shown to increase the hypothalamic-pituitary-adrenal (HPA) axis to promote the release of corticosterone in mice, it stimulates liver gluconeogenesis to regulate energy metabolism [81]. Last but not least, FGF21 functions as a key regulator of the body to adapt to various stress processes [63]. A large amount of evidence shows that increased FGF21 expression occurs as a result of various types of stress. It may be chemical stress induced by acetaminophen, dioxin, or phenylephrine. A similar effect is caused by mitochondrial and oxidative stress, as well as by environmental factors such as cold, starvation, and overfeeding [63].

### 3.2. Biological Effects of FGF21 in the Pathological State

In addition to its important physiological effects, FGF21 also plays an important role in various pathological states. Elevated serum FGF21 levels are considered a compensatory response in the early stages of various metabolic diseases [82–87]. Therefore, FGF21, regarded as the response hormone of stress, is a marker that appears in the early stage of the disease. Several studies have shown an association between FGF21 and diabetes [88, 89]. Elevated FGF21 levels in patients with type 2 diabetes were positively correlated with hypertension, hyperglycemia, glycated hemoglobin, insulin resistance, and high-sensitivity C-reactive protein levels [90]. Due to the function of lowering plasma glucose and lipid levels, FGF21 has been suggested as a potential therapeutic agent for diabetes, obesity, and dyslipidemia [77, 91–94]. FGF21 works as a protective factor in cardiovascular diseases such as atherosclerosis, cardiovascular endothelial injury caused by oxidative stress, and diabetic heart disease [95]. Previous research has proved that FGF21 prevents palmitate-induced cardiac H9c2 cells and primary cardiomyocyte apoptosis by activating the ERK1/2-p38 MAPK-AMPK pathway [96]. Similarly, it also protects the heart muscle cells to slow the development of diabetic heart disease: FGF21 prevents apoptosis in the early stages of cardiac dysfunction and fibrosis in the end stage [96]. FGF21 protects myocardial ischemia-reperfusion injury through the reduction of miR-145-mediated autophagy [97]. In a mouse model of nicotine- (VDN-) induced vascular calcification (VC), exogenous FGF21, *via* the alleviation of endoplasmic reticulum stress- (ERS-) mediated apoptosis, prevents aortic calcification, alleviates the elevation of blood pressure, and ameliorates related injury in VC rats, thereby inhibiting the progression of VC [98]. Partially dependent on PPAR*γ* activity, the FGF21-adiponectin axis is implicated in the control of a variety of metabolic processes [99], which is determined to be a key factor in maintaining cardiovascular homeostasis [100]. Except for protective effects in cardiovascular disease, high FGF21 levels can predict the incidence of coronary artery disease as well [55]. On account of the endocrine manner, FGF21 responds to the injury of other organs in our body. In damaged islets, FGF21 exerts antiapoptotic effects through various mechanisms to maintain *β*-cell function [101]. Animal experiments reveal that FGF21 plays a protective role in the kidney by improving the metabolic system and antifibrotic effect. It is *via* the activation of Akt that FGF21 downregulates TGF-*β*-p53-Smad2/3-mediated epithelial-to-mesenchymal transition, consequently inhibiting diabetes-induced renal fibrosis [102]. A great number of studies have shown the association between CKD and increased circulating FGF21 levels [73, 103, 104]. In a clinical study, plasma FGF21 levels were found to significantly increase with disease progression in patients with chronic and acute kidney disease [105]. In recent years, the effect of FGF21 in lung disease has also received increasing attention. The data shows that FGF21 suppressed inflammation and apoptosis, which provides a possibility for treating lipopolysaccharide-induced acute lung injury [106] *via* the inhibition of the TLR4/Myd88/NF-*κ*B signaling pathway. In pulmonary hypertension, interventional studies show that exogenous FGF21 upregulates PPAR*γ* expression to improve prognosis by AMPK-related pathway [107, 108]. As is widely known, the nuclear factor erythroid2-related factor2 (Nrf-2) signal pathway participates in redox homeostasis [109], which suppresses oxidative stress. In a recent study, researchers observe that FGF21 inhibits pulmonary fibrosis by activating the Nrf-2 pathway, hence inhibiting ECM accumulation, and finally holding back fibrogenesis in pulmonary tissue [10]. The diversiform effects that FGF21 plays in pulmonary disease are still fogged. For example, it is a bird in the hand for researchers to demonstrate that FGF21 functions as a master regulator to have a positive effect on preventing cardiac remodeling, while powerful experimental evidence to ascertain its role in pulmonary remodeling remains insufficient [108]. All these research situations call for further research. Due to the endocrine and autocrine secretion patterns of FGF21, it can act on almost all tissues and organs of the body and play a therapeutic role in diseases of multiple organs. In the latest studies, FGF21 has come to the fore as an emerging therapeutic target for nonalcoholic steatohepatitis and related metabolic diseases [110–114]. Researchers have gradually paid attention to its therapeutic effect, and FGF21 has become a potential therapeutic target for a growing number of diseases, providing a direction for the treatment of more diseases.

In summary, previous studies have demonstrated that FGF21 is a vital regulator of glycolipid metabolism and energy metabolism and also participates in stress response. It not only shows a significant regulatory function in the healthy human body but also plays a unique protective role in the occurrence and development of various diseases. FGF21 is a potential therapeutic target for a growing number of diseases, meanwhile, a beacon tower for some diseases in early stage, including fibrosis.

## 4. Research Progress of FGF21 in Antifibrotic Process

### 4.1. Hepatic Fibrosis

FGF21 has been extensively studied in liver fibrosis-related diseases. Compared with other organs, the function of regulating bile acids, glucose, and lipid metabolism of FGF21 is of prime importance in improving liver fibrosis. Nonalcoholic fatty liver disease (NAFLD) is a series of liver diseases ranging from simple steatosis to nonalcoholic steatohepatitis (NASH) and eventually cirrhosis [115]. Dietary manipulations can induce NAFLD development by using obesogenic or nutrient-deficient diets. Dietary/metabolic model: mice fed with high fructose, high fat, and high cholesterol (diet-induced obesity) are standard animal models for studying NAFLD [67]. Ample evidence shows that the use of exogenous FGF21 can reduce the degree of liver fibrosis in metabolic model mice [116, 117]. *In vivo*, FGF21 ameliorated GPR91 and markers of fibrosis, such as alpha-smooth muscle actin (*α*-SMA) and collagen type 1 [118], production in the liver of methionine and choline-deficient diet-induced mice [119]. Interventional studies report beneficial effects of B1344, a long-acting polyethylene glycosylated (PEGylated) FGF21 analogue, on lowering hepatic fibrosis and protecting against the progression of NASH in rodents and nonhuman primates [120]. FGF21 has also shown significant antifibrotic effects in other liver fibrosis models. Carbon tetrachloride (CCl_4_) induced-model is highly associated with retinol metabolism and PPAR*γ* signaling pathway [121–123]. Dimethylnitrosamine-induced liver fibrosis is mainly related to hepatotoxic and immunotoxic mechanism [124]. It is demonstrated that both low and high doses of FGF21 treatment, in the CCl_4_-induced model, notably downregulated the mRNA levels of *α*-SMA and collagen to slow down the process of liver fibrosis [125]. The same phenomenon was found in dimethylnitrosamine-induced model, which provides more supporting evidence for the antifibrotic effect of FGF21 [125]. Genetic haemochromatosis (GH) is an inherited systemic iron metabolism disorder [126–128], characterized by excessive gut iron absorption and visceral iron deposition [129]. Iron overload in the liver causes progressive fibrosis, contributing to cirrhosis and hepatocellular carcinoma, in which GH patients ultimately die from [130, 131]. The latest studies on the effect of FGF21 on liver fibrosis in haemochromatosis and ferroptosis bring good news for GH patients. It is demonstrated that FGF21 is a novel ferroptosis regulator, which inhibits ferroptosis in primary hepatocytes through regulating cellular iron metabolism and redox balance, thus preventing liver damage and fibrosis induced by iron deposition [132]. Intriguingly, the mechanism of such effect is that FGF21 attenuates iron overload-induced ferroptosis by promoting HO-1 ubiquitination and inducing NRF2 expression [132], which is a special antifibrotic mechanism exclusive to iron overload-induced injury.

**Figure 5:**
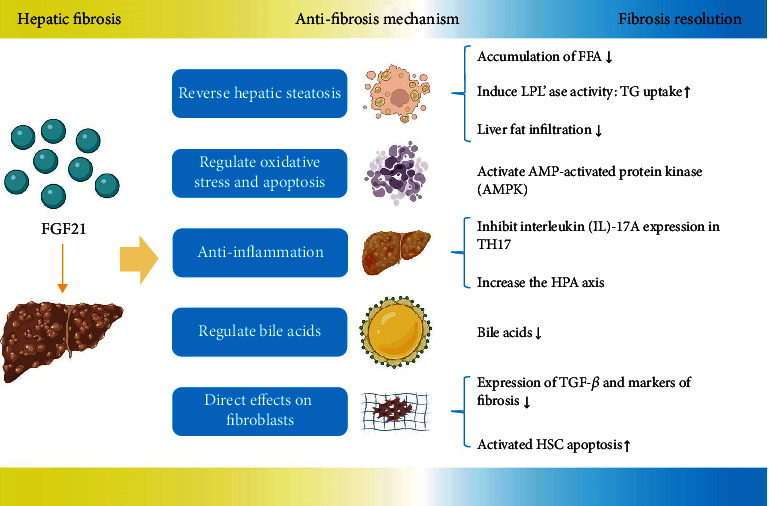
Antifibrotic mechanisms of FGF21 against hepatic fibrosis. The common antifibrotic mechanisms in the liver of FGF21 are divided into five sorts. It attains the target of fibrosis resolution through reversing hepatic steatosis, regulating oxidative stress and apoptosis, anti-inflammatory effects, regulating bile acids, and direct effects on fibroblasts. LPL'ase: lipoprotein lipase; FFA: free fatty acid; HPA axis: hypothalamus-pituitary-adrenocortical axis; TH17: T helper 17; HSC: hematopoietic stem cell.

In existing studies, the common antifibrotic mechanisms in the liver of FGF21 are as follows (Figure 5).

1. Reverse hepatic steatosis: FGF21 has been reported to inhibit lipolysis from the adipose tissue preventing an excess accumulation of FFA in the liver [133]. Furthermore, FGF21 has been shown to increase triglyceride uptake in the adipose tissue by inducing LPL'ase activity [74]. As recently reported, targeting the FGF21/FGFR/*β*-Klotho pathway has the possibility to prevent or reverse liver fat infiltration, inflammation, and fibrosis [14]
2. Regulation of oxidative stress and apoptosis: AMPK in adipocytes and hepatocytes has been described as a mechanism to prevent hepatocyte apoptosis and reduce ERS in NASH [134]
3. Anti-inflammatory effects: the anti-inflammatory effect of an FGF21 analogue has been shown to be mediated *via* the inhibition of interleukin- (IL-) 17A expression in proinflammatory T helper 17 (Th17). Similar to the mechanism in the heart, it is concerned about the adiponectin axis [7]. FGF21 reduces the levels of inflammatory factors (IL-1*β*, IL-6, and TNF-*α*) significantly, achieving therapeutic effects on liver fibrosis, underling the TGF-*β* signaling pathway [125]. Moreover, as FGF21 has been shown to increase the HPA axis in mice, the reaction of increased plasma corticosterone contributes to the anti-inflammatory effect [135]
4. Regulation of bile acids: as a consensus, excess bile acids are toxic and cause liver damage [136]. Exogenous supraphysiological doses of FGF21 have been described to interact with the FGFR4/KLB system, and FGF21 has been shown to decrease Cyp7A1 and bile acids in preclinical models [137]
5. Direct effects on fibroblasts: human stellate cell LX-2 is an activated human HSC model for *in vitro* study of liver fibrosis. Surprisingly, direct antifibrotic actions of FGF21 have been observed in human LX-2 cells [67]. After FGF-21 treatment on dimethylnitrosamine treated mice, the expression of TGF-*β* and fibrosis markers decreased, indicating successful inhibition of HSCs activation [138, 139]. In addition, through upregulating the expression of Caspase-3 and suppressing the ratio of Bcl-2 to Bax, FGF-21 treatment leads to activated HSC apoptosis [138]. A recently published study reveals that FGF21 inhibits HSC proliferation and sensitizes HSC to apoptosis in a dose-dependent manner. The submerged mechanism is that FGF21 ameliorates PDGF-BB-induced HSCs fibrogenesis by blocking the PDGF-leptin-hepatic fibrosis axis [125]. In a nutshell, FGF-21 enjoys a unique position in the cirrhosis antifibrotic process because of its effect on HSC, which proved highly crucial

FGF21 deficiency is conducive to the development of inflammation, hepatocyte damage, and fibrosis. Hence, FGF21 and its analogues have emerged as a potential new therapeutic strategy for liver fibrosis [113]. A large number of clinical studies reveal that the severity of hepatic steatosis and fibrosis is positively correlated with serum FGF21 level [140–144]. Thus, serum FGF21 level has been proposed as a biomarker for NAFLD, NASH, and other liver fibrosis diseases.

### 4.2. Myocardial Fibrosis

The global cardiology community recognizes the increasing burden of myocardial fibrosis disease and its poor health outcomes in the general population. Promising results have shown that FGF21 plays a protective role against cardiac fibrosis. In the process of myocardial fibrosis, FGF21 retards the fibrosis progression by regulating glucose and lipid metabolism and alleviating inflammatory response and antioxidative stress [9, 13, 145–148]. Without a doubt, there is plenty of mechanism for FGF21 to play its anti-inflammatory role in fibrosis disease. FGF21 can effectively ameliorate cardiac inflammation and fibrosis after myocardial infarction by regulating FGFR-EGR1 [149], and more profoundly, to improve heart function [150]. Compared with wild-type mice, mRNA levels of proinflammatory factors, such as IL-6 and monocyte chemoattractant protein-1, were markedly increased in *Fgf21*^−/−^ mice [151]. In addition, it has been reported that FGF-21 treatment inhibits myocardial fibrosis *via* the downregulation of TGF-*β* expression, NF-*κ*B nuclear translocation, and the phosphorylation levels of smad2/3 and I*κ*B-*α* [138], which demonstrates FGF21's anti-inflammatory function in the fibrotic process [152, 153]. Exogenous FGF21 alleviates diabetes-induced myocardial fibrosis by promoting Akt phosphorylation and inhibiting the expression of downstream proinflammatory factors [154]. A recent study of FGF21 KO diabetic mice indicates that FGF21 deletion exacerbates diabetes-induced cardiac fibrosis remodeling [155]. It is the TGF-*β*1-Smad2/3-MMP signaling pathway that plays a pivotal role in the formation process of cardiac fibrosis [156–158]. In myocardial infarction mice model, a new discovery shows that FGF21 ameliorates cardiac fibrosis via inhibiting TGF-*β*1-Smad2/3-MMP2/9 signaling pathway [159] and concurrently exerts heart-protecting function [159]. Simultaneously, the antioxidative mechanism is crucial in FGF21's antifibrotic system. Solid evidence has demonstrated that the Nrf2/ARE signaling pathway is one of the critical pathways responding to oxidative stress in cells [160]. Antioxidant enzymes downstream of the ARE pathway in the Nrf2 compartment include catalase, superoxide dismutase (SOD), and GSH-px [161, 162]. Further studies reported that the administration of FGF21 in diabetic mice led to a significant decrease in ROS levels. Meanwhile, n-Nrf2 increased dramatically and c-Nrf2 was distinctly reduced so that myocardial antioxidant activity was enhanced [153]. As is extensively acknowledged, the mitochondrial oxidative stress pathway performs a pivotal role in cardiomyocytes' apoptosis. Apart from direct antioxidative effects, FGF21 protects cardiomyocytes by regulating the mitochondrial redox system and reducing excessive oxidation [163]. Moreover, PPAR*α* agonist was described to prevent fibrosis in the heart of type 1 diabetic mice (T1DM) *via* the FGF21/PPAR/Sirt1 signaling pathway [9]. The opposite has been observed in mice lacking FGF21. In a hypertension model induced by Ang II treatment for one week, *Fgf21*^−/−^ mice developed a greater degree of extensive cardiac dysfunction and fibrosis [145]. In conclusion, these facts suggest that FGF21 therapy could play a protective role in the development of myocardial fibrosis. Aside from laboratory data, clinical research reveals the indicative potential of FGF21 in myocardial fibrosis as well [164]. Clinical studies have noticed that FGF21 expression is increased in myocardial fibrosis patients [147]. It signals that FGF21 may serve as a brand-new biomarker in evaluating myocardial fibrosis.

### 4.3. Renal Fibrosis

The emerging protective role of FGF21 in renal fibrosis has been widely acknowledged. In chronic kidney fibrosis progression, FGF21 performs its preventive effects and potential therapeutic effects [165–167]. A variety of studies on diabetic mouse models have proved this fact. It has been ascertained that FGF21 suppresses TGF-*β*-p53-Smad2/3-mediated EMT to attenuate diabetes-induced renal fibrosis *via* the activation of Akt [102]. OVE26 transgenic mouse is a widely recommended mouse model to recapture T1DM nephropathy [165]. After 3-month FGF21 treatment, severe renal dysfunction, morphological changes, inflammation, apoptosis, and fibrosis observed in OVE26 mice were significantly attenuated, indicating, for the first time, that FGF21 induces a therapeutic effect on diabetic nephropathy in OVE26 mice [165]. A similar phenomenon was observed in renal fibrosis and chronic renal impairment due to vascular calcification [168]. The latest research shows that FGF21 benefits CKD by cutting off the vicious circle between VC and kidney injury [168]. Beyond that, activating the AMPK-SIRT1-PGC1*α* pathway to inhibit NF-*κ*B function promises FGF21's antifibrotic role [165]. It has been widely admitted that growth factors act as a significant driving force in the pathogenesis of renal fibrosis [166]. Encouragingly, recent studies reveal the participation of FGF21 in regulating growth factors. FGF21 treatments significantly decreased PDGF, VEGF, and CTGF expression, which conspicuously increased in renal fibrosis, by downregulating the phosphorylation level of STAT5 [166]. Above all, it is unquestionable that FGF21 is of extraordinary significance in the prevention and potential treatment of renal fibrosis. Similar to the discovery in the liver and heart, serum levels of FGF21 might be a long-term prognosis indicator of CKD patients. A lower baseline serum level of FGF-21 predicts a better long-term prognosis [169].

### 4.4. Pulmonary Fibrosis

Compared with the organs above, the antifibrotic mechanism of FGF21 in the lung, hitherto, still needs further study. Exogenous FGF21 has been observed to inhibit pulmonary fibrosis. FGF21 attenuates EMT in alveolar epithelial cells and paraquat-treated A549 cells [10]. Further study shows significant downregulation of TGF-*β*, Col I, and *α*-SMA and upregulation of E-cadherin [10]. Based on these results, it has been confirmed that FGF21 inhibits ECM deposition and subsequently pulmonary fibrogenesis. At the same time, FGF21 inhibits fibrosis *via* the Nrf-2 pathway to suppress oxidative stress. It markedly reversed the activity of SOD and T-AOC and decreased the enhanced content of malondialdehyde in the lungs of bleomycin-treated mice [10]. Interventional studies show that exogenous FGF21 attenuates hypoxia-induced inflammatory cytokine secretion [108] through enhancing PPAR*γ* expression in rats, which alleviates hypoxia-induced pulmonary arterial remodeling and collagen deposition *in vivo* [170]. In human pulmonary artery endothelial cells, FGF21 can reduce hypoxia-induced ERS by enhancing PPAR*γ* to prevent the development of fibrosis as well [8]. All in all, FGF21's anti-inflammatory, reducing oxidative stress, and inhibiting EMT mechanism in pulmonary fibrosis have been unearthed by diligent researchers. However, the studies about regulating energy, glucose, and lipid homeostasis, direct effects on myofibroblasts, and other mechanisms are still hazy and limited. These may become the research directions of the mechanism of FGF21 in pulmonary fibrosis in the future.

In conclusion, the crucial preventive and therapeutic role of FGF21 in fibrosis of the liver, heart, kidney, and lung is beyond doubt. It can slow or reverse the fibrosis process by regulating glucose and lipid metabolism, anti-inflammation, antioxidation, antiapoptosis, and inhibiting fibroblast activation. It is expected to be a novel therapeutic target for fibrosis. More specific antifibrotic mechanisms of FGF21 are awaiting discovery.

## 5. Summary and Prospect

Up to now, as a potent pleiotropic regulator of glucose and lipid metabolism, FGF21 has attracted attention from researchers. Simultaneously, it embodies its great importance in the antifibrotic process for the past few years. Sufficient evidence has proved its vital role in the fibrosis of tissues and organs caused by liver, heart, and renal diseases. It acts as a clue to arouse the enthusiasm of the scientists to explore antifibrotic effects in other organs and the antifibrotic mechanism of FGF21. In the occurrence and development of fibrosis, FGF21 not only plays a preventive role but also works as a therapeutic factor in reducing oxidative stress injury, suppressing inflammation, and consequently reversing fibrosis. Since the increased level of serum FGF21 in clinical studies has been found to be correlated with the degree of fibrosis and prognosis of fibrotic disease, FGF21 is expected to become a new biomarker for the evaluation of fibrosis diseases and provide a reference for clinical diagnosis and treatment. Our knowledge about the specific mechanism of FGF21 running in fibrosis continues to be incomplete. Hitherto, the secret of FGF21 targeting the mechanism of antifibrosis is ceaselessly in positive exploration. Therapies based on FGF21 are still relatively new, and their rich biological effects have yet to be fully utilized to treat human diseases. Given this, it is hopefully expected that meaningful novel advances and clinical applications of FGF21 will emerge in further study.

## Abbreviations

AMPK: AMP-activated protein kinase
Ang II: Angiotensin II
CCl_4_: Carbon tetrachloride
CNS: Central nervous system
CKD: Chronic kidney disease
ECM: Extracellular matrix
EMT: Epithelial-to-mesenchymal transition
ERS: Endoplasmic reticulum stress
FFA: Free fatty acid
FGF21: Fibroblast growth factor 21
FGF21-KO: FGF21-knockout
GH: Genetic haemochromatosis
HPA axis: Hypothalamus-pituitary-adrenocortical axis
HSC: Hematopoietic stem cell
LPL'ase: Lipoprotein lipase
MMPs: Matrix metalloproteinases
NAFLD: Nonalcoholic fatty liver disease
NASH: Nonalcoholic steatohepatitis
PPAR*α*: Peroxisome proliferator-activated receptors *α*
SOD: Superoxide dismutase
TH17: T helper 17
VC: Vascular calcification
*α*-SMA: Alpha-smooth muscle actin.
